# Autophosphorylation-based Calcium (Ca^2+^) Sensitivity Priming and Ca^2+^/Calmodulin Inhibition of *Arabidopsis thaliana* Ca^2+^-dependent Protein Kinase 28 (CPK28)*[Fn FN3]^♦^

**DOI:** 10.1074/jbc.M116.763243

**Published:** 2017-01-30

**Authors:** Kyle W. Bender, R. Kevin Blackburn, Jacqueline Monaghan, Paul Derbyshire, Frank L. H. Menke, Cyril Zipfel, Michael B. Goshe, Raymond E. Zielinski, Steven C. Huber

**Affiliations:** From the ‡ Department of Plant Biology, University of Illinois, Urbana, Illinois 61801,; the § Department of Molecular and Structural Biochemistry, North Carolina State University, Raleigh, North Carolina 27695,; ¶ The Sainsbury Laboratory, Norwich NR4 7UH, United Kingdom, and; the ‖ United States Department of Agriculture, Agricultural Research Service, Urbana, Illinois 61801

**Keywords:** Arabidopsis thaliana, autophosphorylation, calcium, calmodulin (CaM), protein-protein interaction, Ca2+-dependent protein kinase

## Abstract

Plant calcium (Ca^2+^)-dependent protein kinases (CPKs) represent the primary Ca^2+^-dependent protein kinase activities in plant systems. CPKs are composed of a dual specificity (Ser/Thr and Tyr) kinase domain tethered to a calmodulin-like domain (CLD) via an autoinhibitory junction (J). Although regulation of CPKs by Ca^2+^ has been extensively studied, the contribution of autophosphorylation in controlling CPK activity is less well understood. Furthermore, whether calmodulin (CaM) contributes to CPK regulation, as is the case for Ca^2+^/CaM-dependent protein kinases outside the plant lineage, remains an open question. We therefore screened a subset of plant CPKs for CaM binding and found that CPK28 is a high affinity Ca^2+^/CaM-binding protein. Using synthetic peptides and native gel electrophoresis, we coarsely mapped the CaM-binding domain to a site within the CPK28 J domain that overlaps with the known site of intramolecular interaction between the J domain and the CLD. Peptide kinase activity of fully dephosphorylated CPK28 was Ca^2+^-responsive and was inhibited by Ca^2+^/CaM. Using *in situ* autophosphorylated protein, we expand on the known set of CPK28 autophosphorylation sites, and we demonstrate that, unexpectedly, autophosphorylated CPK28 had enhanced kinase activity at physiological concentrations of Ca^2+^ compared with the dephosphorylated protein, suggesting that autophosphorylation functions to prime CPK28 for Ca^2+^ activation and might also allow CPK28 to remain active when Ca^2+^ levels are low. Furthermore, CPK28 autophosphorylation substantially reduced sensitivity of the kinase to Ca^2+^/CaM inhibition. Overall, our analyses uncover new complexities in the control of CPK28 and provide mechanistic support for Ca^2+^ signaling specificity through Ca^2+^ sensor priming.

## Introduction

Adaptation to a dynamic environment is necessary for survival and reproduction of all organisms. At the cellular level, environmental stimuli are perceived and coordinated into appropriate cellular responses by highly interconnected signal transduction networks. Following perception by cells, signals are transduced to downstream components by a variety of mechanisms often including activation of second messenger systems such as calcium (Ca^2+^) influxes or via phosphorylation/dephosphorylation cascades involving protein kinases and phosphatases, respectively. Indeed, Ca^2+^ signaling and phosphorylation cascades are among the most well understood mechanisms of communication at the cellular level. The importance of both Ca^2+^ and protein phosphorylation in plant signal transduction is underscored by the considerable expansion of protein families involved in these processes. In the model plant *Arabidopsis thaliana*, the protein kinases are represented by greater than 1000 members (compared with ∼500 in humans) (1), and more than 200 proteins with domains implicated in the sensing of cytosolic Ca^2+^ have been identified (2, 3).

At the confluence of Ca^2+^ signaling and protein phosphorylation lie the Ca^2+^-dependent protein kinases (abbreviated CPKs^4^ in plants and CDPKs in protists). Unique to plants and some protists, CPKs are related to metazoan Ca^2+^/calmodulin (CaM)-dependent protein kinases (CaMKs) and represent an ancestral fusion of a CaMK and CaM (4). CPKs are composed of a variable N-terminal domain implicated in substrate binding (5), a dual specificity Ser/Thr and Tyr protein kinase domain (6), an autoinhibitory junction (J), and a CaM-like Ca^2+^-binding domain (CLD) with four or fewer conserved EF-hand motifs (7). Collectively, the J and CLD form a distinct Ca^2+^-sensing domain known as the CDPK activation domain (CAD) (8). At basal cytosolic Ca^2+^ (∼100 nm), the J domain α-helix extends from the base of the kinase domain, resulting in occlusion of the catalytic cleft and autoinhibition of the kinase (9–12). Under resting conditions, the Ca^2+^-loaded C-terminal EF-hand pair of the CAD interacts with a short sequence within the J domain, stabilizing interaction of the autoinhibitor with the kinase domain (13). Ca^2+^-dependent activation of CPKs is linked to Ca^2+^ binding by the N-terminal EF-hand pair of the CAD (12). Binding of Ca^2+^ by the N-terminal EF-hands leads to dramatic conformational rearrangement of the CAD, resulting in displacement of the pseudosubstrate from the active site and activation of the protein kinase (13). This simple relief of autoinhibition model for CPK activation suggests that removal of the CAD would be sufficient to generate an auto-active kinase domain, and this indeed appears to be the case for some plant CPKs (5). In contrast, a truncation mutant of CDPK1 from *Toxoplasma gondii* (Tg) lacking its CAD is kinase-inactive (14), indicating a more complex role for the CAD. Structural studies of TgCDPK1 reveal an allosteric mechanism where rotation of the CAD and its intramolecular interaction with the kinase domain stabilize the active conformation of the kinase (13–15). Further analysis of the activation mechanism of plant CPKs is required to resolve these conflicting observations and to determine whether the allosteric activation model is applicable to plant CPK isoforms.

As is typical of Arg-Asp (RD)-type protein kinases, CPKs autophosphorylate on Ser and Thr residues. More recently, autophosphorylation on Tyr has been documented for at least four CPKs (*Arabidopsis* CPK4, CPK28, CPK34, and soybean CDPKβ) (6, 16). Although many autophosphorylation sites have been identified for CPKs (16, 17), relatively little is known about the functional consequences of these phosphorylation events. Limited evidence suggests that autophosphorylation enhances CPK activity (18, 19); however, site-specific autophosphorylation might also be inhibitory (6, 19, 20). In the case of Tyr autophosphorylation, the sole study describing functional analysis indicates an inhibitory effect of autophosphorylation at a single Tyr site (6). Thus, it is difficult to draw general conclusions regarding an overall effect of autophosphorylation on CPK activity, and it is likely that site-specific autophosphorylation will affect different properties of CPKs not limited to substrate specificity, subcellular localization, and Ca^2+^-binding/sensitivity.

We are interested in understanding how Ca^2+^, autophosphorylation, and protein-protein interactions contribute to regulating CPK activity. In particular, previous studies (21) prompted us to explore the possibility that CPKs might be regulated by CaM. In this study, we used recombinant CPKs and a number of biochemical approaches to address a possible role for CaM in regulation of CPKs and to gain insight into how autophosphorylation contributes to CPK function. Our analysis identified CPK28 as a novel CaM-regulated protein. Using an *Escherichia coli* protein expression platform enabling differential *in situ* CPK28 autophosphorylation, we assessed the effect of autophosphorylation on substrate phosphorylation using a peptide substrate, and we expanded on the repertoire of known CPK28 autophosphorylation sites. Collectively, our analyses suggest complex regulation of CPK28 by Ca^2+^, autophosphorylation, and CaM binding.

## Results

### 

#### 

##### Recombinant Arabidopsis CPK28 Is a High Affinity CaM-binding Protein

**FIGURE 1.**
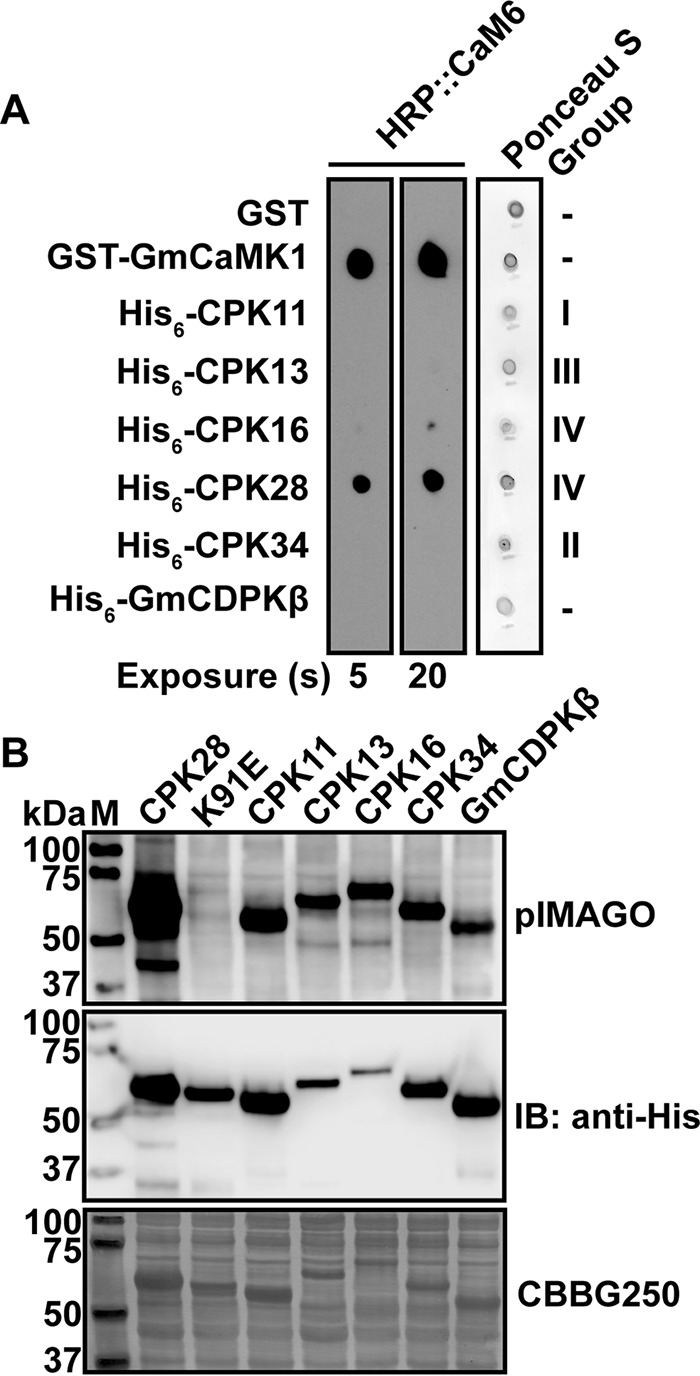
**Analysis of HRP::CaM6 binding to recombinant CPKs.** *A*, spot blot overlay assay of purified His-tagged CPKs along with negative (GST) and positive (GST-GmCaMK1) controls. Nitrocellulose membranes were stained with Ponceau S to assess loading, and the same membrane was probed with 200 nm HRP::CaM6 in the presence of 2 mm CaCl_2_. Binding was detected as described under “Experimental Procedures.” Two different film exposures show that only CPK28 binds substantially at 200 nm HRP::CaM6. Subgroups to which each CPK belong are indicated. *B*, crude extracts containing His-tagged recombinant CPKs were probed for phosphoproteins by blotting with pIMAGO. Anti-His immunoblotting indicates the relative amount of each recombinant kinase. The K91E mutant of CPK28 is shown as a non-phosphorylated control. All recombinant kinases were active and autophosphorylated *in situ. M,* molecular weight marker; *Gm, Glycine max*; *HRP*, horseradish peroxidase; *IB*, immunoblot; *CBB*, Coomassie Brilliant Blue.

The rather surprising observation that some CPKs can interact with CaM (21) prompted us to test whether other members of the *Arabidopsis* CPK family could interact with CaM. Using purified recombinant proteins, we tested binding of CaM6 (a conserved CaM isoform) to a variety of CPKs from different sub-groups in *Arabidopsis* (CPKs 11, 13, 16, 28, and 34) or soybean (GmCDPKβ). Purified recombinant proteins were spotted on nitrocellulose membrane along with glutathione *S*-transferase (GST; negative control) and GST-GmCaMK1, a known CaM-binding protein kinase (22), and membranes were probed for CaM binding using horseradish peroxidase-labeled CaM (HRP::CaM6). We chose 200 nm as a probe concentration because the large number of CaM-binding proteins *in vivo* necessitates high affinity of CaM binding to its targets, and 200 nm is well within the range of dissociation constants described for CaM-binding proteins (23). Under these conditions, we observed binding to GST-GmCaMK1, His_6_-CPK28, and to a lesser extent His_6_-CPK16 but no other CPKs or the GST negative controls (Fig. 1). Importantly, all CPKs were active kinases as evidenced by their ability to autophosphorylate *in situ* (Fig. 1*B*), indicating that recombinant proteins used in binding studies were properly folded. The apparent specific binding of HRP::CaM6 with CPK28 identifies CPK28 as a novel CaM-binding protein (CaMBP). We thus characterized the CPK28-CaM interaction in further detail.

**FIGURE 2.**
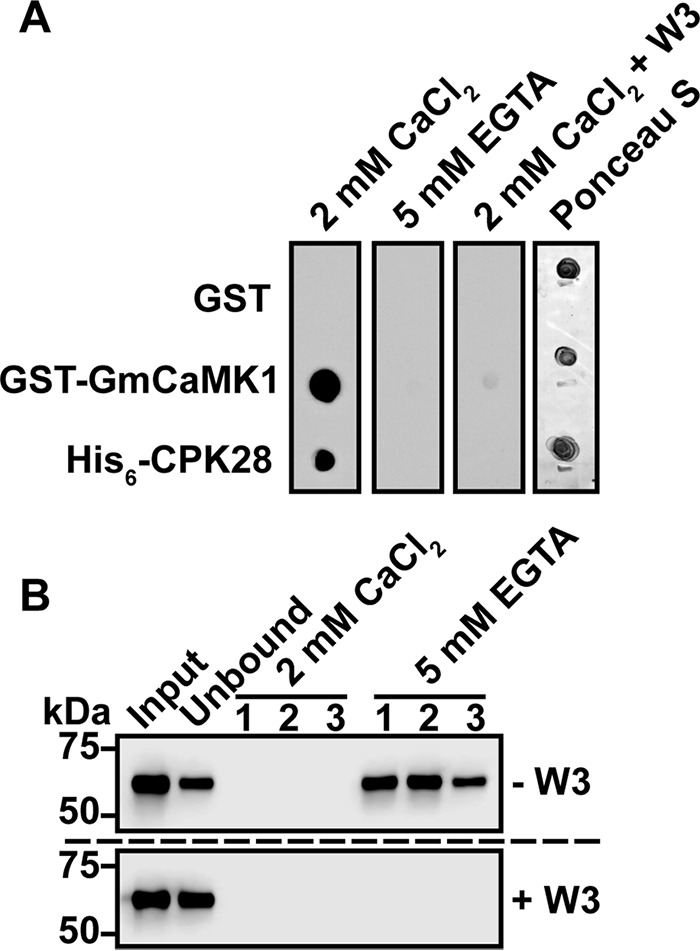
**Interaction between CPK28 and CaM is Ca^2+^-dependent.** *A*, spot blot overlay assay of purified His_6_-CPK28 probed in the presence (2 mm CaCl_2_) or absence (5 mm EGTA) of Ca^2+^ or in the presence of Ca^2+^ with addition of the high affinity CaM-binding peptide W3 (*2 mm CaCl_2_* + *W3*). *B*, binding of CPK28 to immobilized CaM (CaM-Sepharose). 20 μg of purified His_6_-CPK28 was bound to immobilized CaM in the presence of Ca^2+^ (*2 mm CaCl_2_*) with (+*W3*) or without (−*W3*) the addition of the W3 peptide. After washing three times with buffer containing 2 mm CaCl_2_, bound proteins were eluted in three fractions with buffer containing 5 mm EGTA. Fractions were assessed by immunoblotting with anti-His_6_ antibodies.

Many CaM-target interactions occur in a Ca^2+^-dependent manner (23), so we tested Ca^2+^ dependence of the CaM-CPK28 interaction in overlay assays. Interaction of HRP::CaM6 with His_6_-CPK28 was observed only when spot blots were probed in the presence of Ca^2+^; no binding was detected when spot blots were probed in the absence of Ca^2+^ (5 mm EGTA; Fig. 2*A*). To further confirm the specificity of the interaction between HRP::CaM6 and His_6_-CPK28, we incubated spot blots with HRP::CaM6 in the presence of Ca^2+^ and an excess of a high affinity CaM-binding peptide, W3 (24). When blots were probed under these conditions (Fig. 2*A*), we did not observe binding of HRP::CaM6 to His_6_-CPK28, indicating that the signal observed in spot blot overlay assays in the presence of Ca^2+^ was indeed the result of CaM6 interacting with CPK28, rather than nonspecific interaction with the HRP::CaM6 probe.

To confirm overlay results, we used CaM-affinity chromatography as an additional approach to test the interaction between CaM and CPK28. We bound His_6_-CPK28 to CaM-Sepharose in the presence of saturating (2 mm) Ca^2+^ and eluted bound protein with buffer containing 5 mm EGTA. Fractions were then analyzed for the presence of His_6_-CPK28 by immunoblotting with anti-His_6_ antibodies. When His_6_-CPK28 was incubated with CaM-Sepharose in the presence of Ca^2+^, we observed a reduction of immunoblot signal in the unbound *versus* the input fraction indicating that CaM-Sepharose could reduce the amount of His_6_-CPK28 in solution (Fig. 2*B*, *upper panel*). Furthermore, we observed immunoblot signal in EGTA elution but not CaCl_2_ wash fractions (Fig. 2*B*, *upper panel*), collectively indicating that His_6_-CPK28 bound to CaM-Sepharose in a Ca^2+^-dependent manner. When His_6_-CPK28 was incubated with CaM-Sepharose in the presence of the W3 peptide, we did not observe a reduction of immunoblot signal in unbound *versus* input lanes, nor did we observe immunoblot signal in the EGTA elution fractions (Fig. 2*B*, *lower panel*) indicating that His_6_-CPK28 was specifically interacting with immobilized CaM rather than non-specifically with the Sepharose support. Collectively, spot blot overlay and CaM-affinity chromatography experiments indicate that CPK28 is a Ca^2+^-dependent CaM-binding protein.

**FIGURE 3.**
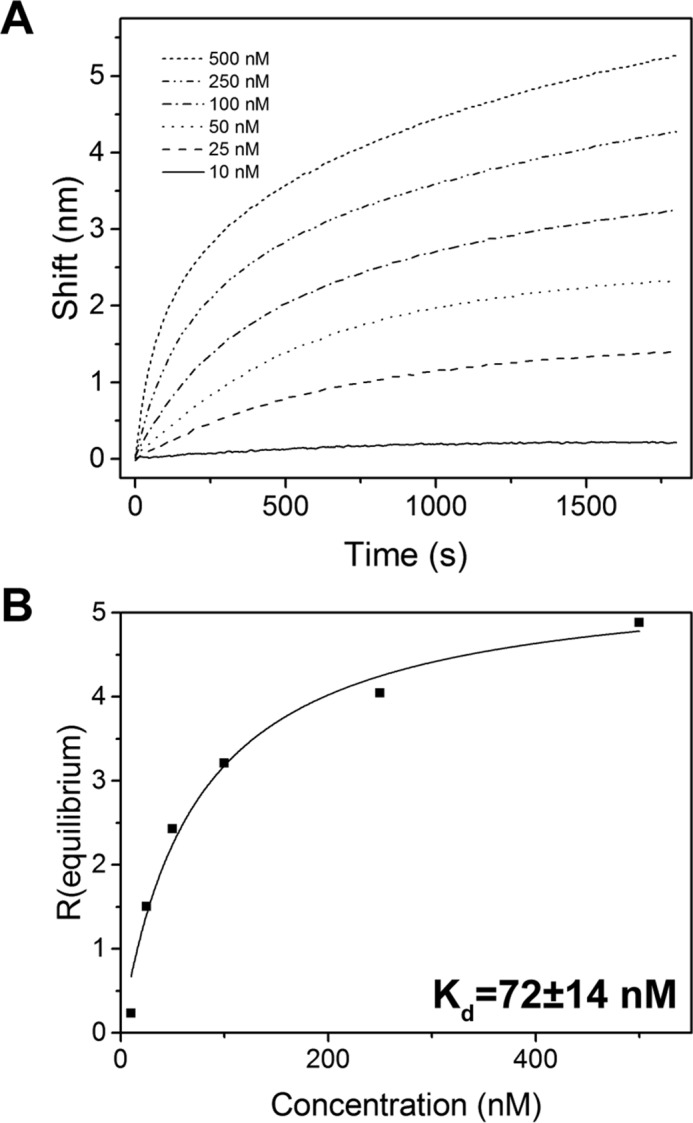
**Kinetic analysis of the CPK28-CaM interaction by biolayer interferometry.** *A*, wavelength shift curves for CPK28 binding to biotin-CaM6 in the Octet system. Biotin-CaM6 was immobilized on streptavidin sensors and was incubated for 30 min (1800 s) with the indicated concentrations of His_6_-CPK28 in the presence of 100 μm CaCl_2_. *B*, determination of a dissociation constant for the CPK28-CaM interaction. *R*_equilibrium_ was determined for each concentration of His_6_-CPK28 (*black squares*) and was fitted using a 1:1 binding model (*black line*) as described under “Experimental Procedures.” The *K_d_* computed from fitted data is shown (±S.E.). Data in *A* and *B* are representative of three replicate experiments performed with independently prepared samples of recombinant CPK28.

The large number of CaM-binding proteins in plant cells necessitates high affinity binding between CaM and its target proteins. Using biolayer interferometry, we assessed binding of a range of CPK28 concentrations to CaM6 immobilized on sensor tips. Binding of CPK28 to immobilized biotinylated-CaM6 occurred with apparent slow kinetics (Fig. 3*A*). Equilibrium binding response (*R*_equilibrium_) values for each concentration of His_6_-CPK28 determined from a 1:1 association binding model were used for the steady-state *K_d_* calculation (Fig. 3*B*). The calculated *K_d_* of the CPK28-CaM interaction was 72 ± 14 nm. Importantly, this value falls well within the range of known CaM targets indicating that the CPK28-CaM interaction could occur under physiological conditions.

To test for *in vivo* association between CPK28 and CaM, we carried out co-immunopurification experiments using *Arabidopsis* plants overexpressing yellow fluorescent protein-tagged CPK28 (CPK28-YFP). As a control, we used stable transgenics expressing the plasma membrane-resident LTI6b protein C-terminally tagged with green fluorescent protein (LTI6b-GFP) similar to previous studies (25, 26). LC-MS/MS analysis indicated that CaM was represented among proteins co-immunopurifying with both CPK28-YFP and the kinase-dead D188A variant (27) but not in LTI6b-GFP samples (see supplemental data). Identification of CaM peptides in co-immunopurification samples for both the wild-type and D188A variants of CPK28 suggests that kinase activity may not be required for CaM binding. We identified two peptides corresponding to residues 92–106 and 107–122 of CaM (Table 1). All four conserved CaM isoforms share 100% sequence identity within these two peptides, and thus we are unable to determine whether a specific isoform of conserved CaM is interacting with CPK28 *in vivo*. Nevertheless, our experiments establish physiological relevance of the CPK28-CaM interaction.

**TABLE 1:** **Calmodulin peptides identified in CPK28-YFP co-immunopurification experiments**

Peptide*^a^*	Sample*^b^*	*M*_r_ (experimental)	Δ (ppm)	Peptide ID probability (%)
R.VFDKDQNGFISAAELR.H	CPK28-YFP	1808.91	2.5	97.3
R.VFDKDQNGFISAAELR.H	CPK28-YFP	1808.91	1.3	99.7
R.VFDKDQNGFISAAELR.H	CPK28-YFP	1808.91	1.3	99.7
R.VFDKDQNGFISAAELR.H	CPK28-YFP	1808.91	2.5	99.7
R.VFDKDQNGFISAAELR.H	CPK28-YFP	1808.91	0.9	99.7
R.VFDKDQNGFISAAELR.H	CPK28-YFP	1808.91	0.9	95.9
R.VFDKDQNGFISAAELR.H	CPK28-YFP	1808.91	0.9	99.7
K.(ox)MKDTDSEEELKEAFR.V	CPK28^D188A^-YFP	1842.83	1.3	98.9
K.(ox)MKDTDSEEELKEAFR.V	CPK28^D188A^-YFP	1842.83	0.3	99.0
K.(ox)MKDTDSEEELKEAFR.V	CPK28^D188A^-YFP	1842.83	0.3	99.0
K.(ox)MKDTDSEEELKEAFR.V	CPK28^D188A^-YFP	1842.83	1.3	99.7
R.VFDKDQNGFISAAELR.H	CPK28^D188A^-YFP	1808.91	2.1	99.7
R.VFDKDQNGFISAAELR.H	CPK28^D188A^-YFP	1808.91	1.9	99.7
R.VFDKDQNGFISAAELR.H	CPK28^D188A^-YFP	1808.91	1.9	99.7

*^a^* (ox)M represents oxidized methionine.

*^b^* Overexpression of CPK28-YFP or CPK28^D188A^-YFP is in the *cpk28-1* knockout background.

##### CaM Binds a CPK28 Junction Domain Peptide

**FIGURE 4.**
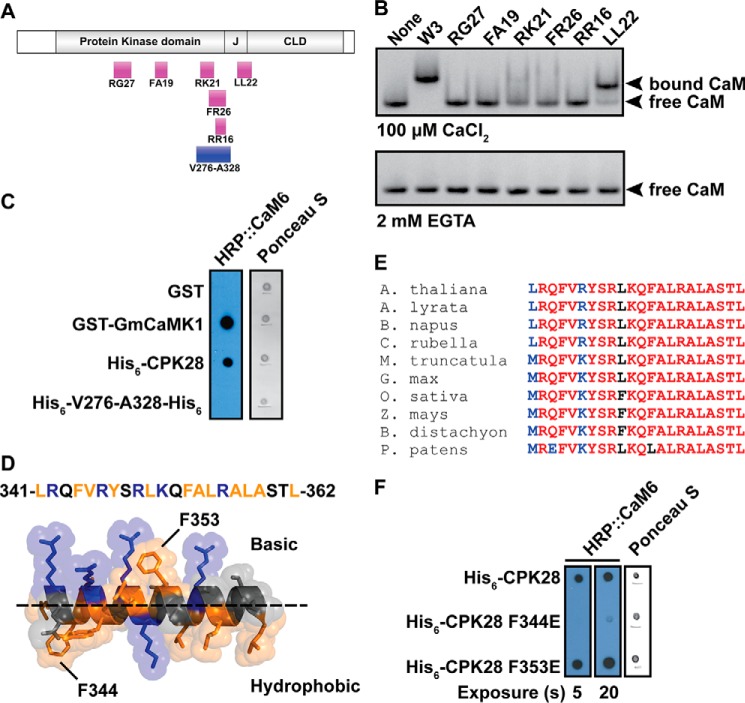
**CaM binds to a peptide from the CPK28 junction domain.** *A*, schematic map of the domain organization of CPK28 showing the location of peptides (*magenta boxes*) and recombinant proteins (*blue boxes*) used to delineate the CaM-binding domain. *B*, peptide binding by CaM6 in native PAGE in the presence (100 μm CaCl_2_) or absence (2 mm EGTA) of Ca^2+^ as indicated. CaM-peptide complexes were visualized by staining with GelCode Blue protein stain after electrophoresis. *C*, overlay assay indicating lack of binding to a C-terminal fragment (Val-276 to Ala-328) of the CPK28 kinase domain. Proteins were spotted to nitrocellulose membrane and probed with 200 nm HRP::CaM6. Full-length CPK28 was detected by HRP::CaM6 but the Val-276 to Ala-328 fragment was not. *D*, helical model of the LL22 peptide rendered in PyMOL. Basic residues are shown in *blue,* and hydrophobic residues are shown in *orange*. Basic and hydrophobic faces of the amphipathic helix are indicated (above and below the *dashed line*, respectively). Potential hydrophobic anchor residues are indicated. *E*, HRP::CaM6 overlay analysis of CaM binding to CPK28 CaMBD mutants. Approximately 400 ng of each protein was spotted, and the blot was probed with 100 nm HRP::CaM6. *CaMBD*, CaM-binding domain; *HRP,* horseradish peroxidase. *F*, Clustal-Omega alignment of the CPK28 CaM-binding domain with the corresponding region from other species. The CaM-binding domain is highly conserved across diverse plant taxa. GenBank^TM^ accession numbers for sequences used for the alignment are: *Arabidopsis lyrata*, XP_002865081.1; *Brassica napus*, XP_013653730.1; *Capsela rubella*, XP_006282144.1; *Medicago truncatula*, XP_003612167.1; *Glycine max*, XP_003538879.1; *Oryza sativa*, NP_001059444.1; *Zea mays*, NP_001151048.1; *Brachypodium distachyon*, XP_003557300.1; *Physcomitrella patens*, XP_001766209.1.

Using a combination of *in silico* prediction and peptide binding experiments, we identified a CaM-binding domain at the C-terminal end of the CPK28 autoinhibitory junction. This approach was adopted after attempts to produce truncated forms of recombinant CPK28 proved unsuccessful due to susceptibility of truncated proteins to proteolysis during expression in *E. coli*. We interrogated the CPK28 primary sequence for CaM-binding sites using the Calmodulin Target Database and identified a number of putative CaM-binding domains (CaMBD; Fig. 4*A*). To determine which of these sites interacted with CaM, we obtained synthetic peptides corresponding to each putative site, and we tested CaM binding by native PAGE using the W3 peptide as a positive control. We observed an electrophoretic mobility shift of CaM with the W3 peptide and the LL22 peptide derived from the CPK28 autoinhibitory junction (Fig. 4*B*), indicating that the LL22 peptide was bound by CaM. No other peptides derived from CPK28 induced a similar mobility shift for CaM. Furthermore, the mobility shift only occurred in the presence of Ca^2+^ (Fig. 4*B*), confirming Ca^2+^ dependence of the CPK28-CaM interaction. In addition to the synthetic peptides tested in native PAGE assays, we generated a recombinant protein consisting of residues Val-276 to Ala-328 of CPK28 spanning the combined region represented by the RK21 and FR26 peptides derived from the C-terminal end of the kinase domain. This recombinant protein did not interact with HRP::CaM6 in overlay assays (Fig. 4*C*), and thus we conclude that this region does not confer CaM binding to full-length CPK28. Modeling of the LL22 peptide (Fig. 4*D*) indicates that the binding site is amphipathic in nature, as is typical for CaMBDs. This region is highly conserved among higher plant species (Fig. 4*E*) suggesting that CaM binding might have been retained by CPK28 orthologs during evolution of the plant CPK lineage.

The putative binding domain contains two bulky hydrophobic residues (Phe-344 and Phe-353), which may serve as anchors for CaM binding. We generated recombinant proteins harboring non-conservative Phe-to-Glu mutations for each of these residues (F344E and F353E) and tested their ability to bind CaM in overlay assays. The CPK28 F344E mutant was almost completely null for HRP::CaM6 binding (Fig. 4*F*) at a probe concentration of 100 nm, suggesting that Phe-344 may serve as an anchor residue for CaM interaction with CPK28. By comparison, the F353E mutant was unaffected with regard to CaM binding under these conditions. Importantly, loss of binding to the CPK28 F344E mutant provides additional support for the likely CaM-binding site lying within the CPK28 J domain.

##### CaM Binding Inhibits CPK28 Autophosphorylation and Peptide Kinase Activity

**FIGURE 5.**
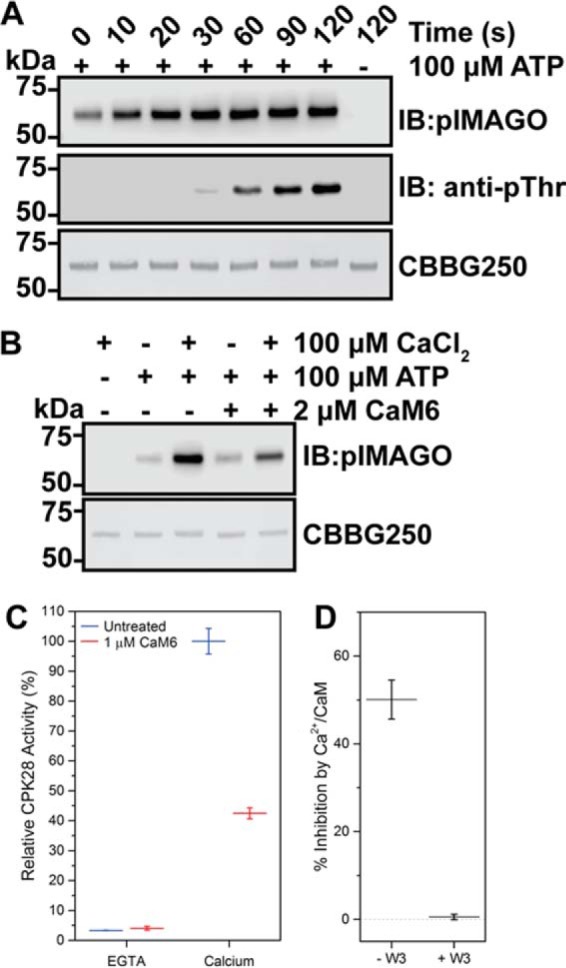
**CaM inhibits Ca^2+^-activated autophosphorylation and transphosphorylation of fully dephosphorylated CPK28.** *A*, time-dependent autophosphorylation of fully dephosphorylated His_6_-CPK28. A single reaction was initiated by the addition of ATP and was sampled at the time points indicated. A reaction lacking ATP was included as a control. Samples were assessed by blotting with the pIMAGO reagent or with anti-phosphothreonine (*anti-pThr*) antibodies. Approximately 500 ng of protein was loaded in each lane. *B*, effect of Ca^2+^ and Ca^2+^/CaM on autophosphorylation of CPK28. Reactions were prepared as indicated, initiated by the addition of ATP, and allowed to proceed for 30 s. Samples were assessed by blotting with the pIMAGO reagent, and a CBBG250-stained membrane is shown to demonstrate equal loading (∼500 ng) for each sample. *C*, effect of Ca^2+^ and Ca^2+^/CaM on CPK28 peptide kinase activity. CPK28 activity toward the ACSM+1 peptide (10 μm) was assessed in the absence (EGTA) or presence (calcium) of 100 μm CaCl_2_ with or without the addition of 1 μm CaM6 as indicated. Values reported are CPK28 activity relative to the Ca^2+^-activated state. *D*, W3 peptide blocks Ca^2+^/CaM inhibition of CPK28 peptide kinase activity. Activity of Ca^2+^-activated CPK28 was assessed in the absence (− *W3*) or presence (+ *W3*) of 2 μm W3 peptide, with or without the addition of 1 μm CaM6 as indicated. Specific activities under each condition tested are shown (*inset*). Point estimates in *C* and *D* represent mean with standard deviation of three technical replicates. Data shown are representative of at least two independent preparations of recombinant CPK28. *IB,* immunoblot; *CBBG250*, Coomassie Brilliant Blue G-250.

To determine the functional consequences of CaM binding to CPK28, we carried out a series of *in vitro* kinase assays using fully dephosphorylated recombinant CPK28 (see “Experimental Procedures”). CPK28 autophosphorylated rapidly *in vitro* in an ATP-dependent manner (Fig. 5*A*), and this activity was enhanced by the addition of Ca^2+^ (Fig. 5*B*). When CPK28 was autophosphorylated in the presence of Ca^2+^/CaM, we observed a reduction in pIMAGO signal (Fig. 5*B*), indicating reduced autophosphorylation of the kinase. Similarly, we tested the effect of Ca^2+^ and Ca^2+^/CaM on activity of CPK28 toward a synthetic peptide substrate derived from tomato 1-aminocyclopropane-1-carboxylate synthase (ACSM+1; NNLRLSMGKR). Activity of recombinant CPK28 toward the ACSM+1 peptide was strictly Ca^2+^-dependent as indicated by near-complete inhibition of peptide kinase activity by EGTA (Fig. 5*C*). In the presence of Ca^2+^/CaM, peptide kinase activity of recombinant CPK28 was 43% (95% CI: 38, 47) of the Ca^2+^-stimulated activity (Fig. 5*C*), indicating that binding of Ca^2+^/CaM to CPK28 resulted in inhibition of CPK28 peptide kinase activity. One simple explanation for the observed reduction of CPK28 activity in the presence of CaM is competition for Ca^2+^ between CaM and CPK28 in our kinase activity assays. To rule out this possibility, we tested the ability of CaM to inhibit CPK28 peptide kinase activity in the absence or presence of the W3 peptide. In the absence of W3, we observed typical inhibition of CPK28 kinase activity as in other experiments (Fig. 5*D*). Importantly, the W3 peptide on its own was not inhibitory to CPK28 activity; specific activity in the presence of W3 was 7.5 pmol/min/μg kinase compared with 6.5 pmol/min/μg kinase in the absence of W3. By comparison, when the W3 peptide was included in the kinase assay, Ca^2+^/CaM inhibition of CPK28 activity was abolished (Fig. 5*D*) indicating that inclusion of CaM in the kinase reactions is not simply reducing the amount of Ca^2+^ available to CPK28. Collectively, experiments indicate that binding of CaM to the CPK28 J domain inhibits both auto- and transphosphorylation *in vitro* thereby establishing regulatory function of the Ca^2+^/CaM-CPK28 interaction.

**FIGURE 6.**
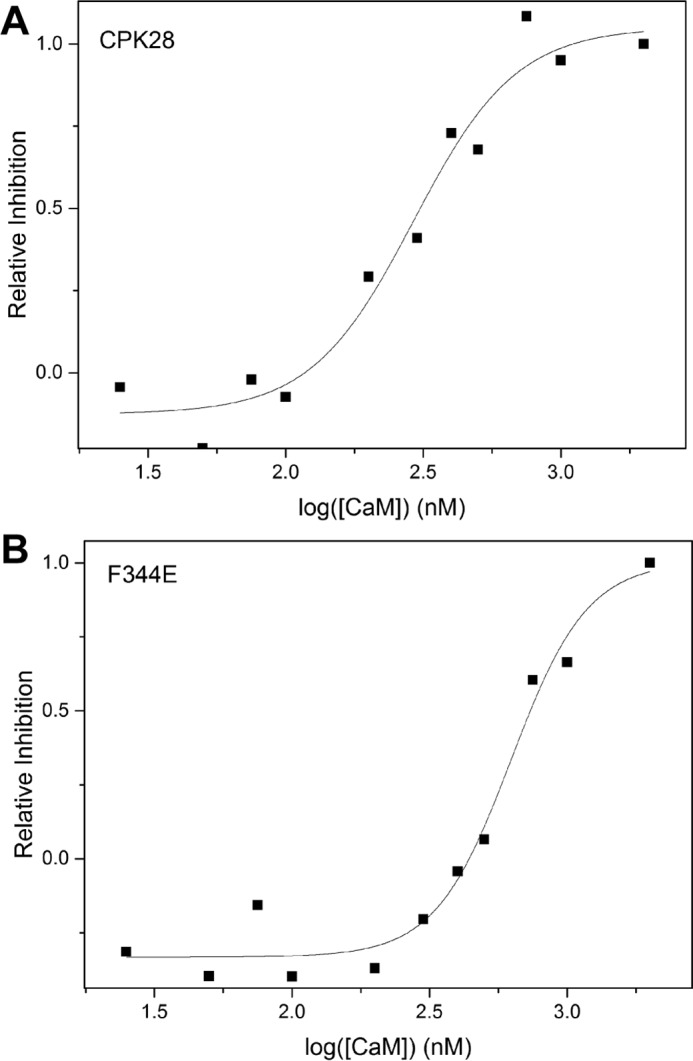
**Ca^2+^/CaM-inhibition of wild-type CPK28 and the F344E mutant.** Representative determinations of the IC_50_ for Ca^2+^/CaM inhibition of CPK28 (*A*) or the F344E mutant (*B*). Assays were performed at 100 μm CaCl_2_, 100 μm ATP, and 10 μm ACSM+1 peptide. CaM inhibition is shown on a relative scale where 100% inhibition corresponds to an ∼60% loss of activity compared with untreated protein.

Additionally, we tested the effect of CaM on kinase activity of the CPK28 F344E CaM-binding mutant. Activity of the wild-type kinase and the CPK28 F344E mutant toward the ACSM+1 peptide was assessed across a range of CaM concentrations to estimate an IC_50_ for CaM. The IC_50_ for CaM inhibition of wild-type CPK28 ranged from 291 to 348 nm. By comparison, IC_50_ values for CPK28 F344E ranged from 525 to 626 nm (Table 2; Fig. 6, *A* and *B*) indicating that the CPK28 F344E mutant is less sensitive to CaM compared with the wild-type protein. In addition to reduced CaM sensitivity, specific activity of the CPK28 F344E was ∼40% of the wild-type protein (Table 2).

**TABLE 2:** **Kinase activity comparison of recombinant CPK28 and the F344ECaM-binding mutant**

	CPK28	F344E
Specific activity (pmol·min^−1^·μg^−1^ kinase)*^a,b^*	9.3 ± 1.5	4.0 ± 1.2
CaM sensitivity		
Replicate no. 1		
IC_50_ (nm)	291 ± 39	575 ± 86
Adjusted *R*^2^	0.952	0.940
Replicate no. 2		
IC_50_ (nm)	348 ± 84	525 ± 82
Adjusted *R*^2^	0.909	0.941
Replicate no. 3		
IC_50_ (nm)	327 ± 41	626 ± 61
Adjusted *R*^2^	0.951	0.962

*^a^* Data were assessed using 0.5 μg of kinase, 100 μm ATP, and 10 μm ACSM+1 peptide in the presence of 100 μm CaCl_2_.

*^b^* Mean is with a 95% CI.

##### Differential in Situ Autophosphorylation and Phosphosite Profiling of CPK28

**FIGURE 7.**
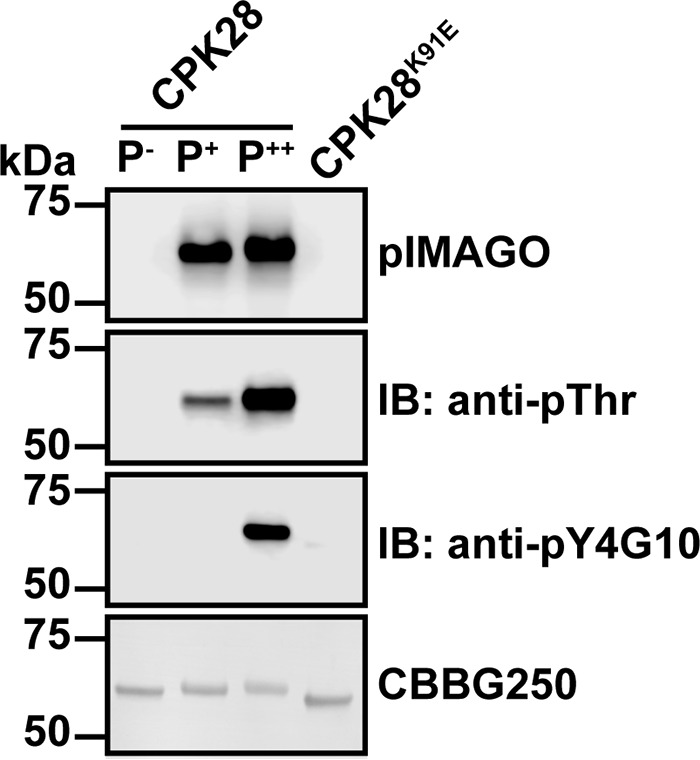
**Production of differentially phosphorylated forms of CPK28 during recombinant protein production.** Proteins were expressed under three different conditions as described under “Experimental Procedures.” Autophosphorylation status of each phosphoform (P^−^, P^+^, and P^++^) was assessed by blotting with pIMAGO and, anti-Thr(P) and anti-Tyr(P) antibodies as indicated. The K91E kinase-dead mutant is shown as a non-phosphorylated control. Approximately 500 ng of protein was loaded in each lane. *IB,* immunoblot; *CBBG250,* Coomassie Brilliant Blue G-250.

As is the case for many Ser/Thr protein kinases, CPKs autophosphorylate on Ser and Thr residues, and it is now recognized that CPKs are capable of Tyr autophosphorylation as well (6, 16). Previously, we used *in situ* autophosphorylated protein to generate autophosphorylation profiles for several members of the leucine-rich repeat receptor-like kinase family (28), and we carried out similar experiments for CPK28 in this study. We established expression conditions in *E. coli* to produce differentially autophosphorylated forms (“phosphoforms”) of CPK28. Recombinant CPK28 was produced under three different conditions (see “Experimental Procedures”), and autophosphorylation of the different preparations was assessed by immunoblotting using the kinase-dead K91E variant as a control to rule out possible activity of endogenous *E. coli* protein kinases (Fig. 7). CPK28 produced during co-expression with λ protein phosphatase had no detectable autophosphorylation, whereas protein produced without λ protein phosphatase co-expression had low or high levels of autophosphorylation when induction was carried out in the absence or presence of 5 mm CaCl_2_, respectively (Fig. 7). We refer to these three phosphoforms as P^−^-CPK28 (dephosphorylated), P^+^-CPK28 (low phosphorylation), and P^++^-CPK28 (high phosphorylation). It is noteworthy that only the P^++^-CPK28 phosphoform was immunoreactive with anti-phosphotyrosine antibodies, indicating that elevated [Ca^2+^]_cyt_ may be required for CPK28 to achieve dual specificity.

We subjected the P^+^- and P^++^-CPK28 phosphoforms to trypsin digestion and analysis by LC-MS/MS to identify specific sites of autophosphorylation (Table 3). Our analysis identified all previously observed CPK28 autophosphorylation sites with the exception of Ser-213 in the activation loop as has been reported previously (16). Importantly, our experiments confirm autophosphorylation on Tyr-463 in EF-loop III of the CAD. In addition to known autophosphorylation sites, we identified eight novel autophosphorylation sites distributed among all subdomains of CPK28.

**TABLE 3:** **Phosphopeptides identified from *in situ* autophosphorylated recombinant CPK28** Data shown are from representative peptide identifications; all product ion spectra were inspected manually to ensure correct phosphosite identification.

Site*^a^*	*M*_r_ (experimental)	*M*_r_ (calculated)	E-value	Peptide	Fragmentation	Ref.
**Ser-6**	988.4201	988.4202	0.0041	M.GVCF**pS**AIR.V	HCD	
Ser-24	1207.5859	1207.5849	4.2e-07	K.**pS**KAAPTPIDTK.A	HCD	16
Thr-33	1379.6712	1379.6697	0.00032	K.AAPTPID**pT**KASTK.R	HCD	16
Ser-36	1379.6718	1379.6697	0.00095	K.AAPTPIDTKA**pS**TK.R	HCD	16
**Thr-33/Thr-37**	1615.7376	1615.7372	0.00043	K.AAPTPID**pT**KAS**pT**K.R	ETD	
Ser-43	898.3620	898.3620	0.0014	R.TG**pS**IPCGK.R	HCD	16
**Ser-198**	1037.4808	1037.4794	1.0e-05	K.**pS**AQLDSPLK.A	HCD	
Ser-228	1986.9549	1986.9492	5.1e-10	R.FHDIVG**pS**AYYVAPEVLK.R	HCD	16, 17, 27
Ser-318	1502.7392	1502.7395	6.0e-05	R.LTAAQAL**pS**HAWR.E	HCD	16, 17, 27
**Thr-329**	2075.9707	2075.9888	0.0016	R.EGGNA**pT**DIPVDISVLNNLR.Q	HCD	
**Ser-360**	1682.7788	1682.7764	9.5e-09	R.ALA**pS**TLDEAEISDLR.D	HCD	
Tyr-463*^b^*	1271.5453	1271.5435	0.0036	K.DG**pY**ITPEELR.M	HCD	16
**Ser-478**	1607.7088	1607.7080	3.9e-06	R.G**pS**IDPLLDEADIDR.D	HCD	-
Ser-495	1280.5914	1280.5914	0.00058	R.DGKI**pS**LHEFR.R	HCD	17, 27
**Ser-510**	928.4015	928.4015	0.0002	R.TASIS**pS**QR.A	HCD	
**Ser-510**	1701.7954	1701.7948	3.0e-10	R.TASIS**pS**QRAPSPAGHR.N	ETD	
**Ser-507/Ser-510**	1008.3676	1008.3678	0.039	R.TApSIS**pS**QR.A	HCD	
**Ser-507/Ser-510**	1781.7618	1781.7611	0.00013	R.TA**pS**IS**pS**QRAPSPAGHR.N	ETD

*^a^* Sites listed in boldface are new sites identified only in this study.

*^b^* Tyr(P)-463 was only identified in samples from protein expressed with the addition of CaCl_2_ to the expression culture at the time of induction.

##### Autophosphorylation Status Affects Ca^2+^ Responsiveness and CaM Sensitivity of CPK28

**FIGURE 8.**
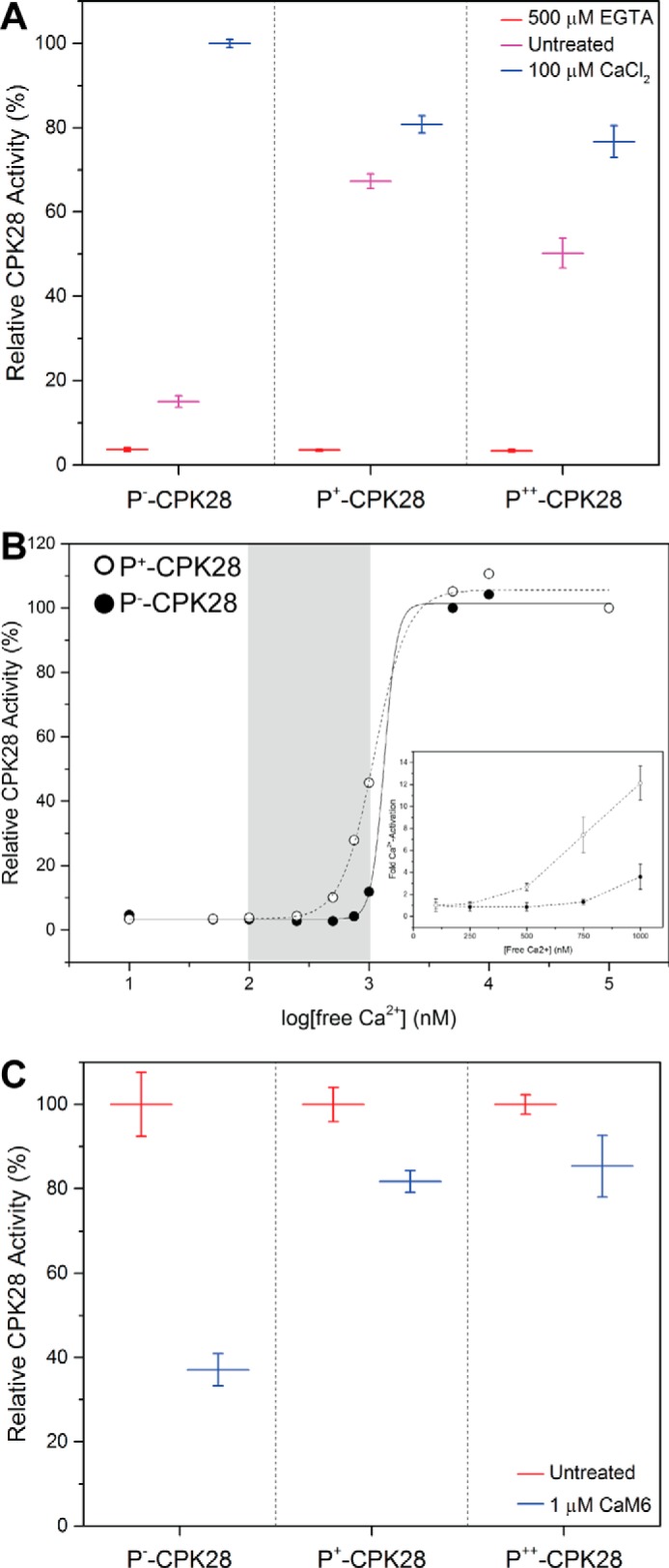
**Autophosphorylation enhances basal levels, Ca^2+^ responsiveness, and Ca^2+^/CaM sensitivity of CPK28 peptide kinase activity.** *A*, peptide kinase activity of the three different CPK28 phosphoforms toward the ACSM+1 peptide substrate (10 μm). Activity was measured in the presence of 500 μm EGTA, 100 μm CaCl_2_, or no treatment (*Untreated*). Activities are reported relative to Ca^2+^-stimulated P^−^-CPK28. *B*, analysis of P^−^- and P^+^-CPK28 peptide kinase activity across a range of free Ca^2+^ concentrations. *Open circles,* P^+^-CPK28; *closed circles,* P^−^-CPK28. *Gray* region represents the physiological range of free Ca^2+^ in plants (∼100 to 1000 nm); *inset*, fold activation of P^−^-CPK28 and P^+^-CPK28 by Ca^2+^ across the physiological range. Fold activation was calculated relative to the activity at 100 nm free Ca^2+^. Point estimates are mean with 95% confidence intervals. *C*, peptide kinase activity of the three different CPK28 phosphoforms in the presence of 100 μm CaCl_2_ with or without (*untreated*) the addition of 1 μm CaM6. For each phosphoform, activity is reported relative to the untreated control. Point estimates in *A* and *C* represent means with standard deviation of three technical replicates. Data shown are representative of at least two independent preparations of recombinant CPK28.

We assessed the ability of the CPK28 phosphoforms to phosphorylate the ACSM+1 peptide substrate under different conditions of available Ca^2+^. Activity of recombinant CPK28 phosphoforms was assessed in the presence of excess Ca^2+^ (100 μm CaCl_2_), the complete absence of Ca^2+^ (500 μm EGTA), or without treatment of any kind (untreated, hereafter referred to as “background Ca^2+^”). All three CPK28 phosphoforms were sensitive to inhibition by EGTA (Fig. 8*A*), indicating strict requirement for Ca^2+^ regardless of autophosphorylation status. P^−^-CPK28 had low activity at background Ca^2+^ and showed ∼6-fold stimulation by excess (100 μm) Ca^2+^. Surprisingly, both the P^+^- and P^++^-CPK28 phosphoforms had enhanced activity at background Ca^2+^ compared with P^−^-CPK28 (4.5- and 3.3-fold enhancement, respectively), and neither was substantially stimulated by addition of 100 μm CaCl_2_ (Fig. 8*A*). Furthermore, both P^+^- and P^++^-CPK28 displayed a modest reduction in maximum activity compared with P^−^-CPK28 (Fig. 8*A*), indicating that autophosphorylation might also have a small inhibitory effect on substrate phosphorylation by CPK28 at high levels of Ca^2+^. To better understand how autophosphorylation effects Ca^2+^ activation of CPK28, we assessed peptide kinase activity at different concentrations of free Ca^2+^ spanning below and above the physiological range (Fig. 8*B*). Both phosphoforms were Ca^2+^-activated; however, P^+^-CPK28 responded more robustly and at lower concentrations of free Ca^2+^. Relative to kinase activity at 100 nm free Ca^2+^, activation of P^−^-CPK28 could only be detected at 1000 nm free Ca^2+^ (3.5-fold activation). By comparison, Ca^2+^ activation of P^+^-CPK28 could be detected at 500 nm free Ca^2+^, and it was approximately four times greater at 1000 nm free Ca^2+^ (12-fold activation; Fig. 8*B*, *inset*). These results indicate that autophosphorylation “primes” CPK28 for Ca^2+^ activation.

We next tested the effect of Ca^2+^/CaM binding on the activity of each phosphoform toward the ACSM+1 peptide in the presence of Ca^2+^ (Fig. 8*C*). As expected, peptide kinase activity of P^−^-CPK28 was sensitive to CaM (63% inhibition (95% CI, 54, 72)). In contrast, Ca^2+^/CaM inhibition for P^+^- and P^++^-CPK28 was 18% (95% CI, 9, 27) and 14% (95% CI, 5, 23), respectively, indicating that autophosphorylated CPK28 was less sensitive to inhibition by Ca^2+^/CaM compared with the dephosphorylated protein. Collectively, our analysis indicates that CPK28 autophosphorylation modifies Ca^2+^ sensitivity of the kinase and relieves inhibition by Ca^2+^/CaM.

**FIGURE 9.**
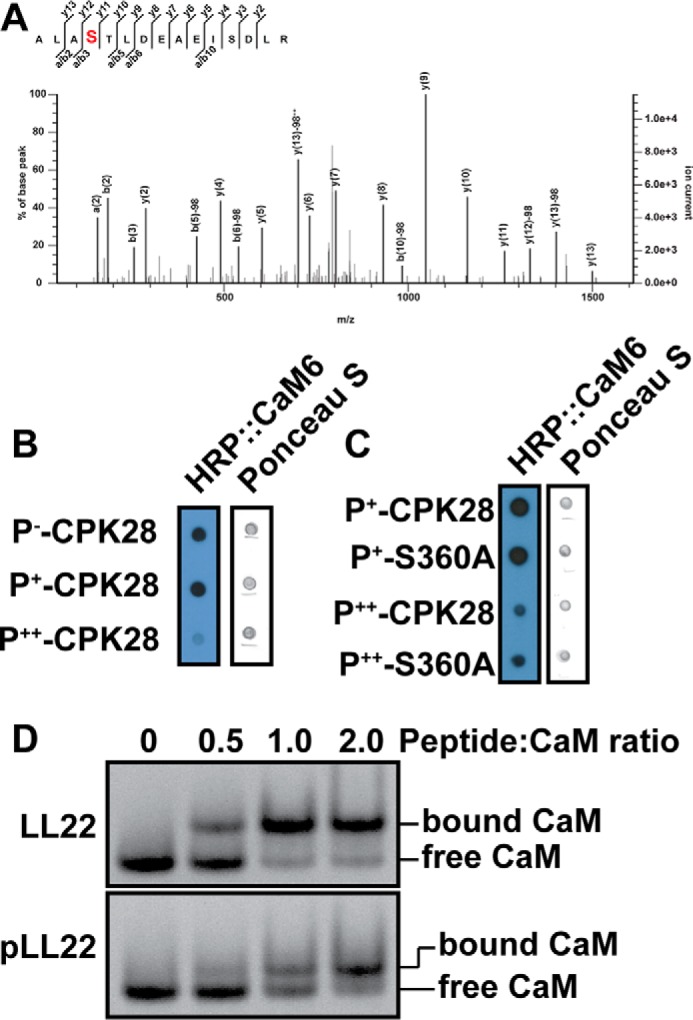
**Effect of Ser-360 autophosphorylation on CaM binding to CPK28.** *A,* product ion spectrum identifying Ser-360 within the CPK28 CaM-binding domain as an autophosphorylation site. *B*, HRP::CaM6 binding to the three CPK28 phosphoforms. Approximately 400 ng of each protein was spotted, and the blot was probed with 100 nm HRP::CaM6 in the presence of calcium (2 mm). *C*, HRP::CaM6 binding for phospho-CPK28 and S360A mutants. Approximately 400 ng of each protein was spotted, and the blot was probed with 100 nm HRP::CaM6. *D*, native PAGE analysis of CaM binding to the LL22 peptide and its phosphorylated variant (pLL22). A near-complete mobility shift of CaM required more pLL22 peptide compared with the dephosphorylated form. CaM (240 pmol) was incubated with different amounts of peptide in the ratios indicated in a 20-μl reaction for 60 min prior to electrophoresis. *HRP*, horseradish peroxidase.

Because our LC-MS/MS experiments identified Ser-360 within the putative CPK28 CaMBD as an autophosphorylation site (Fig. 9*A*), we hypothesized that autophosphorylation might directly inhibit binding of Ca^2+^/CaM to CPK28. We tested binding of HRP::CaM6 to the three CPK28 phosphoforms and to a S360A mutant in overlay assays. We detected reduced binding of the HRP::CaM6 probe to P^++^-CPK28 compared with P^−^- and P^+^-CPK28 (Fig. 9*B*); however, the CPK28 S360A mutant did not restore binding of the probe to P^++^-CPK28 (Fig. 9*C*). We also tested the effect of phosphorylation in native PAGE experiments using a modified LL22 peptide phosphorylated at Ser-360 (pLL22, LRQFVRYSRLKQFALRALA(pS)TL; Fig. 9*D*). CaM bound the LL22 peptide as indicated by reduced mobility of CaM in native PAGE, and binding was saturated at a 1:1 molar ratio of peptide to CaM (Fig. 9*D*). CaM also bound the pLL22 peptide; however, the proportion of CaM with reduced mobility at equimolar CaM and peptide was lower relative to unphosphorylated LL22 (Fig. 9*D*) indicating reduced binding of pLL22 to CaM. Whereas peptide-binding experiments are suggestive of inhibition of CaM binding by phospho-Ser-360, this does not appear to hold true for CaM binding to full-length recombinant protein based on overlay assay results with the CPK28 S360A mutant. Thus, we tentatively conclude that phospho-Ser-360 is not the sole phosphosite mediating reduced CaM binding to P^++^-CPK28 and diminished CaM sensitivity of peptide kinase activity of the P^+^- and P^++^-CPK28 phosphoforms.

## Discussion

Many protein kinases from distinct families are among the known repertoire of plant CaMBPs. Members of the receptor-like cytoplasmic kinase (22), leucine-rich repeat receptor-like kinase (29, 30), CPK-related kinase (CRK) (31), and an S-locus receptor kinase (32) are CaMBPs, in addition to the well known Ca^2+^ and Ca^2+^/CaM-dependent protein kinase involved in bacterial and fungal symbioses (33). Besides those protein kinases characterized in detail, a protein array-based screen (21) for CaM and CaM-like-binding proteins identified an extensive set of protein kinases from multiple families, including the CPKs, as putative CaMBPs, although the majority of these interactions remain to be confirmed by further analysis. The potential for interaction of CaM with CPKs prompted us to test a small number of recombinant CPKs for CaM binding. Previously, it was suggested that CPK3, CPK10, and CPK30 could interact with CaM (21). In this study, we expand on this set of CaM-binding CPKs, identifying CPK28 as a novel CaMBP. CPK28 belongs to subgroup IV of the *Arabidopsis* CPKs that includes CPK16 and CPK18, with which it shares 82 and 72% sequence identity, respectively. Given the relatively high amino acid sequence identity between CPK16 and CPK28, it is interesting that CPK28 uniquely interacted with CaM in overlay assays and is suggestive of subfunctionalization among CPKs. It is noteworthy that subgroup IV of the *Arabidopsis* CPK family is closely related to the CRKs, some of which are CaM-regulated, raising the possibility for evolutionary conservation of CaM binding in some but not all plant protein kinases descended from eukaryotic CaMKs. Like many other CaMBPs, CPK28 binds CaM with high affinity and in a Ca^2+^-dependent manner. CPK28 bound CaM with an ∼70 nm *K_d_*, which is well within the range of known CaM targets (*K_d_* values are typically within the 10 nm to 1 μm range, see Ref. 23) and the physiological levels of CaM. Furthermore, we detected CaM in a complex with CPK28 in co-immunopurification experiments, and although we cannot rule out the possibility that this observation is indirect, these experiments support the notion that CaM and CPK28 could interact under physiological conditions. Our co-immunopurification experiments were not able to distinguish which specific CaM isoform was present in complex with CPK28-YFP due to high sequence identity of conserved CaMs. It seems likely that all four conserved *Arabidopsis* CaM isoforms could interact with CPK28 and other CaM targets; however, differential expression of *CaM* genes (34) might confer greater specificity in binding among CaM and its multitude of interacting partners.

We used an *in silico* approach to identify putative CaM-binding sequences within CPK28. This analysis revealed a large number of putative binding domains within the CPK28 primary structure. Among synthetic peptides designed against these various predicted CaMBDs, only one could be demonstrated empirically to interact with CaM *in vitro*. Predicting CaMBDs is complicated by the fact that CaM targets a conserved secondary structure for binding rather than a primary sequence, and our results suggest a high rate of false-positive identification of CaMBDs *in silico*. We suggest that other authors interpret CaMBD predictions cautiously, and we recommend empirical confirmation *in vitro* before experiments in plants are carried out where it is difficult to assess the functional integrity of mutant proteins that appear not to bind CaM. Delineation of the CPK28 CaMBD revealed a binding site within the J domain in a position homologous to CaMBDs found in animal CaMKs and in plant CRKs. The CPK28 CaMBD is highly conserved among land plant species, suggesting positive selection for CaM binding to CPK28 and its orthologs. Interestingly, the putative CPK28 CaMBD overlaps with the known site of intramolecular interaction between the J and CLD domains of CPKs, suggesting the possible requirement for conformational rearrangement of CPK28 prior to CaM binding. This idea is supported by the relatively slow association kinetics observed in real time binding assays. However, further structural analysis will be required to test this hypothesis. Analysis of the CPK28 F344E mutant, which has reduced CaM binding compared with the wild-type protein, provides additional support for the location of the CPK28 CaMBD. The F353E mutant of CPK28 was unaffected with regard to CaM binding implying that Phe-353 is dispensable for formation of the CaM-CPK28 complex. Importantly, the F344E mutant was less sensitive to Ca^2+^/CaM inhibition in kinase assays but is not a good candidate for *in vivo* functional analysis of the CaM binding to CPK28 because of its reduced specific activity compared with the wild-type protein. Alternative approaches will be required to address the importance of the CPK28-CaM interaction in plants.

Autophosphorylation has been extensively documented for CPKs, but information is lacking regarding the biochemical function and physiological consequences of these events. We manipulated relative autophosphorylation levels of CPK28 using specific conditions for expression of recombinant protein in *E. coli* and showed that autophosphorylation modulates both Ca^2+^ activation and Ca^2+^/CaM sensitivity of the kinase. In agreement with a previous study (27), chelation of Ca^2+^ by treatment with EGTA inhibited kinase activity indicating a strict requirement for bound Ca^2+^. Addition of Ca^2+^ to 100 μm stimulated CPK28 activity compared with background Ca^2+^; however, this was only true for the fully dephosphorylated kinase. Remarkably, autophosphorylated CPK28 was almost fully active at background Ca^2+^ *in vitro* and was no longer responsive to added Ca^2+^. When we analyzed Ca^2+^ activation of dephosphorylated and autophosphorylated CPK28 in more detail, we found that autophosphorylated CPK28 responded more robustly and at a lower concentration of free Ca^2+^ compared with the dephosphorylated kinase. Thus, autophosphorylated CPK28 is “primed” for Ca^2+^ activation and would be activated by a lower threshold of Ca^2+^ influx in plant cells. Furthermore, activity of autophosphorylated CPK28 might decline more slowly relative to dephosphorylated kinase during attenuation of Ca^2+^ signals. The rate of this deactivation phase could be further modified by as yet unknown protein phosphatases acting on CPK28. This regulatory mechanism could arise from an autophosphorylation-based increase in Ca^2+^ affinity of the CAD or from stabilization of the active conformation of the protein, and current work is aimed at addressing this question.

The role of autophosphorylation in control of CPK28 is strikingly similar to regulation of human CaMKII by autophosphorylation following activation by Ca^2+^/CaM. Autophosphorylation of Thr-286 in the CaMKII autoinhibitory domain locks the kinase in the activated state by markedly slowing dissociation of Ca^2+^/CaM upon Ca^2+^ withdrawal, ultimately generating a Ca^2+^-independent enzyme (35–37). CaMKII autophosphorylation thus serves as a form of molecular memory allowing the kinase to “remember” previous Ca^2+^ signaling events via sustained CaMKII activation after autophosphorylation and return to resting [Ca^2+^]_cyt_. Our analysis suggests that CPK28 autophosphorylation may serve a similar function, where autophosphorylation during an initial Ca^2+^ signaling event would generate a form of the kinase primed for subsequent Ca^2+^ influx events. Despite these similarities, autophosphorylation-based control of CPK28 must occur via a distinct mechanism because of structural differences between the kinases, and the observation that CPK28 does not possess an autophosphorylation site directly homologous to the regulatory Thr-286 site of CaMKII. Our observation that autophosphorylation results in priming of CPK28 Ca^2+^-dependent activity provides mechanistic support for the Ca^2+^ sensitivity priming model of signaling specificity in Ca^2+^-mediated pathways. Genetic and Ca^2+^ imaging studies in guard cells reveal that certain stimuli (*e.g.* abscisic acid and CO_2_) enhance the Ca^2+^ sensitivity of stomatal closing responses, indicating that Ca^2+^-dependent pathways for stomatal closure can be primed for activation (38–41). It has been suggested that priming is regulated by the activity of protein phosphatases (41); however, evidence supporting this hypothesis is lacking. We propose that Ca^2+^ sensor phosphorylation, in particular CPK autophosphorylation, might constitute a biochemical signature of the primed state of Ca^2+^-mediated signaling pathways.

From a physiological perspective, CPK28 functions to dampen plant immune responses in the absence of pathogen attack by phosphorylating and contributing to turnover of BOTRYTIS-INDUCED KINASE 1 (BIK1) prior to pathogen-induced Ca^2+^ influx (42, 43), a role that would require that CPK28 be active at low [Ca^2+^]_cyt_. As such, we speculate that site-specific CPK28 autophosphorylation will be a critical regulatory mechanism for control of plant immune homeostasis. Furthermore, Ca^2+^-dependent CaM binding to CPK28 following the rise of [Ca^2+^]_cyt_ during pathogen attack would attenuate Ca^2+^-dependent activation of CPK28, reducing BIK1 turnover and enhancing BIK1-mediated immune signaling. Alternatively, CaM binding to autophosphorylated CPK28 could block further autophosphorylation at specific sites or shield specific sites from the activity of protein phosphatases, functioning as a mediator of autophosphorylation-based control of CPK28. Understanding how autophosphorylation and CaM binding control CPK28 activity *in planta* will require studies detailing spatio-temporal properties of CPK28 autophosphorylation *in vivo* and are outside the scope of this study.

In conclusion, our analysis of recombinant CPK28 reveals previously unknown mechanisms regulating kinase activity, Ca^2+^/CaM binding and modulation of Ca^2+^ sensitivity by autophosphorylation. Importantly, regulation of CPK28 by CaM binding and autophosphorylation fit well into current models of the physiological role of CPK28 in controlling plant immune homeostasis. It will be interesting in the future to see whether autophosphorylation similarly controls other members of the CPK family and how both Ca^2+^/CaM binding and autophosphorylation contribute to regulation of CPK28 in plants.

## Experimental Procedures

### 

#### 

##### Cloning and Constructs

Clones for CPK11, CPK13, and CPK16 were obtained from the *Arabidopsis* Biological Resource Center. The CPK28 cDNA was amplified from a cDNA pool derived from *Arabidopsis* ecotype Col-0 leaf tissue. Oligonucleotide primers used for amplification are listed in Table 4. PCR product was digested with appropriate restriction enzymes, gel-purified, and ligated into digested pET28a(+) for in-frame fusion with an N-terminal His_6_ tag. Clones were isolated and confirmed by DNA sequencing and *in silico* translation. Confirmed clones were transformed into different host strains for recombinant protein expression as described below.

**TABLE 4:** **Oligonucleotide primers used in this study**

Name	Restriction site	Sequence*^a^*	Purpose
CPK28_F_NdeI	NdeI	atcgcatatgGGTGTCTGTTTCTCCGCC	pET28a cloning
CPK28_R_NotI	NotI	atcggcggccgcCTATCGAAGATTCCTGTGACC	pET28a cloning
CPK11_F_NdeI	NdeI	atcgcatatgGAGACGAAGCCAAACCCTAGACG	pET28a cloning
CPK11_R_NotI	NotI	atcggcggccgcTCAGTCATCAGATTTTTCACC	pET28a cloning
CPK13_F_NdeI	NdeI	atcgcatatgGGAAACTGTTGCAGATCTCCCGC	pET28a cloning
CPK13_R_NotI	NotI	atcggcggccgcCTATTCGTTGCCTAGGTTC	pET28a cloning
CPK16_F_NheI	NheI	atcggcatgcATGGGTCTCTGTTTCTCCTCC	pET28a cloning
CPK16_R_NotI	NotI	atcggcggccgcTTAGACCTTGCGAGAAATAAGATAACC	pET28a cloning
S360A		CAATTTGCTTTAAGGGCGCTTGCTgcCACACTTGACGAGGCAGAGATCTC	CPK28 mutagenesis
S360Arc		GAGATCTCTGCCTCGTCAAGTGTGgcAGCAAGCGCCCTTAAAGCAAATTG	CPK28 mutagenesis
F344E		CATTTCAGTTCTGAACAACTTAAGACAAgaaGTGAGATACAGCCGTCTAAAGC	CPK28 mutagenesis
F344Erc		GCTTTAGACGGCTGTATCTCACttcTTGTCTTAAGTTGTTCAGAACTGAAATG	CPK28 mutagenesis
F353E		GAGATACAGCCGTCTAAAGCAAgaaGCTTTAAGGGCGCTTGCTAGC	CPK28 mutagenesis
F353Erc		GCTAGCAAGCGCCCTTAAAGCttcTTGCTTTAGACGGCTGTATCTC	CPK28 mutagenesis

*^a^* Underlined residues deviate from the wild-type CPK28 sequence for directed mutagenesis.

Site-directed mutagenesis of the pET28a(+):CPK28 expression clone was carried out using procedures exactly as described previously (44). Oligonucleotide primers used for site-directed mutagenesis are listed in Table 4. All mutagenized clones were confirmed by DNA sequencing and *in silico* translation prior to transformation into protein expression hosts for production of recombinant protein.

##### Recombinant Protein Expression and Purification

Recombinant CPK28 and site-directed mutants were expressed as N-terminal His_6_-tagged fusion proteins in *E. coli* under various conditions as described below. Expression cultures consisting of Luria broth (LB) containing 50 μg/ml kanamycin were inoculated with 1:100 volume of saturated starter culture and were incubated with shaking at 37 °C until the culture reached an optical density of ∼0.6 at 600 nm. Production of His_6_-CPK28 was induced by adding isopropyl β-d-1-thiogalactopyranoside (IPTG) to a final concentration of 0.5 mm, and expression was allowed to proceed for 16 h at room temperature. To obtain dephosphorylated His_6_-CPK28 (P^−^-CPK28), expression was carried out in the *E. coli* BL21(DE3)-VR2-pACYC-LamP host strain that co-expresses λ protein phosphatase (13). Hyper- and hypo-phosphorylated His_6_-CPK28 (P^++^-CPK28 and P^+^-CPK28) were obtained by expression in T7 Express LysY/I^q^ with or without the addition of 5 mm CaCl_2_ to the culture at the time of induction, respectively. Cells from 500 ml of culture were pelleted at 3500 × *g* for 10 min and were suspended in 25 ml of cell extraction buffer (50 mm Tris-HCl, pH 7.5, containing 0.5 mm 4-(2-aminoethyl)benzenesulfonyl fluoride, 10 μm leupeptin, 1 mm benzamidine hydrochloride, 2 mm ϵ-aminocaproic acid, and 0.5 μm E-64, 10 mm NaF, 1 mm Na_3_VO_4_, and 1 mm Na_2_MoO_4_). Cells were lysed by treatment with 250 μg/ml lysozyme at room temperature for 20 min followed by one freeze-thaw cycle and treatment with 125 units of benzonase nuclease (EMD Millipore) to reduce viscosity. Additional sonication (four 10-s cycles with 10-s rests) was used for lysis of the BL21(DE3)-VR2-pACYC-LamP host. Lysates were clarified by centrifugation at 35,000 × *g* for 30 min. Crude lysates were adjusted to 300 mm NaCl and 50 mm imidazole followed by gravity-flow purification with nickel-nitrilotriacetic acid (Qiagen) exactly as described previously (23). Elution fractions were pooled, concentrated, and dialyzed against three changes of 1000 volumes of 25 mm Tris-HCl, pH 7.5, 100 mm NaCl, 1 mm dithiothreitol (DTT). Protein concentration was typically determined by Bradford assay using bovine serum albumin as standard or by absorbance at 276 nm in 6 m guanidine HCl using a calculated extinction coefficient of 50,880 m^−1^·cm^−1^ (for determination of binding kinetics). Purity was assessed by SDS-PAGE and staining with Coomassie Brilliant Blue G-250 protein stain (Sigma). Aliquots of protein were stored at −80 °C until use or were used immediately for determination of binding kinetics.

Recombinant CaM6 (untagged) was expressed in the *E. coli* NiCO (DE3) (New England Biolabs) host strain. Cultures (LB containing 50 μg/ml ampicillin) were inoculated as described above and were grown at 37 °C to an optical density of ∼0.6 at 600 nm. Protein production was induced by the addition of 0.5 mm IPTG, and expression was allowed to proceed for 4 h at 37 °C. Cells from 100 ml of culture were suspended in 10 ml of cell extraction buffer as described above but lacking NaF, Na_3_VO_4_, and Na_2_MoO_4_. Cells were lysed by treatment with 250 μg/ml lysozyme for 20 min at room temperature followed by a single freeze-thaw cycle and sonication (see above). Debris was pelleted at 35,000 × *g* for 30 min. CaCl_2_ was added to the clarified lysate to a final concentration of 5 mm, and the lysate was heated to 90 °C for 3 min. Precipitated *E. coli* proteins were removed by centrifugation for 20 min at 35,000 × *g*. Recombinant CaM6 was purified as described (45) by Ca^2+^-dependent hydrophobic interaction chromatography using phenyl-Sepharose (GE Healthcare) chromatography resin.

##### SDS-PAGE and Immunoblotting

For SDS-PAGE, proteins were diluted with 4× NuPAGE loading buffer (Life Technologies, Inc.) to a final loading buffer concentration of 1×. SDS-PAGE samples additionally contained 100 mm DTT as reductant. Proteins were separated in 10% (v/v) acrylamide BisTris-HCl, pH 6.8, NuPAGE gels. For immunoblotting experiments, proteins were transferred to polyvinylidene fluoride (PVDF) membrane. Membranes were washed with phosphate-buffered saline (8 mm sodium phosphate, 2 mm potassium phosphate, 137 mm NaCl, 2.7 mm KCl) containing 0.1% (v/v) Tween 20 (PBS-T) and then blocked for 1 h to overnight at room temperature in PBS-T containing 5% (w/v) cold water fish skin gelatin (Sigma). After blocking, membranes were rinsed in PBS-T and were then incubated with appropriate primary antibodies as follows: anti-His_6_ (Sigma product no. H1029, lot no. 013M4866), 1:3000, overnight incubation at room temperature; anti-phosphothreonine (anti-Thr(P); Sigma product no. SAB5200089, lot no. 131122), 1:500, 2-h incubation at room temperature; anti-phosphotyrosine (anti-pY4G10, Millipore product no. 05-1050, lot no. 2475690), 1:1,000, overnight at 4 °C. After primary antibody binding, membranes were washed three times for 5 min each in PBS-T and were then incubated for 1 h at room temperature in either goat anti-rabbit HRP (Thermo Fisher Scientific, product no. 31462, lot number unavailable) or goat anti-mouse HRP (Thermo Fisher Scientific, product no. 31432, lot no. PE1855475) as appropriate at a dilution of 1:20,000 in PBS-T containing 1% (w/v) cold water fish skin gelatin (Sigma). Membranes were washed as above and then rinsed with PBS to remove excess detergent. Blots were then incubated in Pierce ECL Western blotting substrate (Thermo Fisher Scientific) following the manufacturer's instructions, and chemiluminescent signals were captured using a c-Digit blot scanner (Li-Cor Biosciences). For detection of total phosphorylation of recombinant proteins, blotting with the pIMAGO reagent (Tymora Analytical) was carried out exactly according to the manufacturer's instructions except that iodoacetamide was not added to SDS-PAGE samples of purified recombinant proteins. pIMAGO blots were imaged using the Pierce ECL Western blotting substrate and the c-Digit blot scanner.

##### Generation of HRP::CaM6 and Spot Blot Overlay Assays

Purified recombinant CaM6 was reduced at 55 °C for 1 h with 100 mm DTT followed by dialysis against three changes of 1000 volumes of degassed PBS. Reduced CaM6 was combined with maleimide-activated horseradish peroxidase (HRP; Thermo Fisher Scientific) at a 1:1 molar ratio, and the labeling reaction was carried out for 6 h at room temperature. Labeled HRP::CaM6 was purified by Ca^2+^-dependent hydrophobic interaction chromatography to remove unreacted HRP, and the purified probe was assessed by SDS-PAGE. To generate biotinylated CaM6 for Octet assays, 1 mg of purified CaM6 (1 mg/ml concentration) was incubated with 1.2 mmol of biotin-PEG_2_-maleimide (Thermo Fisher Scientific) overnight. Excess labeling reagent was removed by dialysis against 1000 volumes of 25 mm Tris-HCl, pH 7.5. Aliquots of HRP::CaM6 and Biotin-PEG_2_-CaM6 were stored at −80 °C until use.

HRP::CaM6 spot blot assays were carried out by spotting purified recombinant proteins as indicated to nitrocellulose. The amount of protein spotted was typically 0.5 μg unless otherwise indicated. Spots were dried, and membranes were stained with Ponceau S to confirm equal loading of proteins. Membranes were rinsed briefly with 20 ml of Tris-buffered saline (TBS, 25 mm Tris-HCl, pH 7.5, 140 mm NaCl, 2.5 mm KCl) containing 2 mm CaCl_2_ (TBS-C) to remove the Ponceau stain and were then blocked in the same buffer with 5% (w/v) skim milk powder for 1 h at room temperature. Membranes were rinsed briefly with TBS-C to remove excess milk and were then probed with 200 nm HRP::CaM6 in TBS-C containing 1% (w/v) skim milk powder for 1 h at room temperature. Spot blots were washed three times for 10 min each in TBS-C. After washing, membranes were incubated in Pierce ECL Western blotting substrate following the manufacturer's instructions. HyBlot CL autoradiography film (Denville Scientific) was exposed to membranes for 5–20 s and then developed in a Kodak M35A X-OMAT processor. Under these conditions, interaction of HRP::CaM6 with CPK28 and the positive control could be routinely detected with 5-s film exposures. Developed films were digitized using a CanoScan 4400F flatbed scanner (Canon). All His_6_-CPK28 samples for binding experiments were expressed under conditions giving P^+^-CPK28 as described above unless otherwise indicated.

##### Immobilized CaM Chromatography

CaM-Sepharose (GE Healthcare) binding experiments were carried out in 50 mm Tris-HCl, pH 7.5, containing 2 mm CaCl_2_ and 1 mm DTT (binding buffer). 20 μg of His_6_-CPK28 in binding buffer (1-ml final sample volume) was incubated with 40 μl of resin (40-μg binding capacity) for 1 h at room temperature in a microcentrifuge tube on a rotator. After binding, resin was pelleted for 5 min at 2400 × *g*. Resin was washed three times with 1 ml of binding buffer, followed by three elutions (80 μl each) with 50 mm Tris-HCl, pH 7.5, 5 mm EGTA. Equal volumes of each fraction (input, unbound, washes, and elutions) were separated by SDS-PAGE and transferred to PVDF for immunoblotting with anti-His_6_ antibodies as described above.

##### Real Time Protein-Protein Interactions Assays in the Octet System

Quantitative analysis of the CaM6-CPK28 interaction was carried out using biolayer interferometry in the Octet system (Forte Bio). Biotinylated CaM6 (10 μg/ml) prepared as described above was immobilized for 30 s on streptavidin biosensors. After immobilization, signal baseline was determined in TBS-C for 100 s before incubating CaM6-labeled biosensors in different concentrations of purified recombinant His_6_-CPK28 prepared as a serial dilution in TBS-C from a 10 μm stock solution. Association and dissociation steps were carried out for 1800 s in TBS-C. Binding experiments were carried out at 30 °C and 1000 rpm shaking.

Raw binding traces were buffer subtracted and aligned to the baseline reading for individual sensors. Using the association step only, equilibrium binding (*R*_equilibrium_) was determined for each concentration of His_6_-CPK28 by fitting binding traces to a 1:1 binding model in the Octet data analysis software. *R*_equilibrium_ values were plotted against concentration, and *K_d_* was determined using a steady-state binding model as shown in Equation 1,

(Eq. 1) $$R_{\text{equilibrium}}=R_{\max}\left(\frac{\left[\text{CPK}28\right]}{\left[\text{CPK}28\right]+K_{d}}\right)$$

Initial values for *R*_max_ and *K_D_* were estimated at 5 and 100 nm, respectively. Steady-state curve fitting and *K_d_* determination was carried out in OriginPro 2015 (OriginLab Corp.).

##### Immunoprecipitation of CPK28-YFP and Detection of Associated Proteins by LC-MS/MS

Creation of stable transgenic complementing *cpk28–1/35S*::*CPK28-YFP* and non-complementing *cpk28–1/35S*::*CPK28-D188A-YFP* lines was previously described (27). Control Col-0/*LTI6b-GFP* lines were also previously described (25, 26). Immunoprecipitation of LTI6b-GFP, CPK28-YFP, or CPK28-D188A-YFP was performed as described previously (42) using anti-GFP μMACS magnetic beads (Miltenyi Biotec). Proteins were eluted in pre-warmed SDS-PAGE loading buffer per the manufacturer's instructions and subjected to SDS-PAGE. Protein samples were prepared for MS analysis by excising bands from SDS-polyacrylamide gels stained in Coomassie Brilliant Blue (Simply Blue^TM^ Safe stain, Invitrogen). Gel slices were destained in 50% acetonitrile and incubated for 45 min in 10 mm DTT. Cysteinyl residue alkylation was performed for 30 min in the dark in 55 mm chloroacetamide. After several washes with 25 mm ammonium bicarbonate, 50% acetonitrile gel slices were dehydrated in 100% acetonitrile. Gel pieces were rehydrated with 50 mm ammonium bicarbonate and 5% acetonitrile containing 20 ng/μl trypsin (Pierce), and digestion proceeded overnight at 37 °C. Tryptic peptides were sonicated from the gel in 5% formic acid, 50% acetonitrile, and the total extracts were evaporated until dry.

LC-MS/MS analysis was performed using an Orbitrap Fusion trihybrid mass spectrometer (Thermo Fisher Scientific) and a nanoflow-UHPLC system (Dionex Ultimate3000, Thermo Fisher Scientific). Peptides were trapped to a reverse phase trap column (Acclaim PepMap C18, 5 μm, 100 μm × 2 cm, Thermo Fisher Scientific) connected to an analytical column (Acclaim PepMan 100, C18 3 μm, 75 μm × 50 cm, Thermo Fisher Scientific). Peptides were eluted in a gradient of 3–30% acetonitrile in 0.1% formic acid (solvent B) over 50 min followed by gradient of 30–80% B over 6 min at a flow rate of 300 nl/min at 40 °C. The mass spectrometer was operated in positive ion mode with nano-electrospray ion source with an inner diameter of 0.02 mm fused silica emitter (New Objective). Voltage 2200 V was applied via platinum wire held in PEEK T-shaped coupling union with transfer capillary temperature set to 275 °C. The Orbitrap, MS scan resolution of 120,000 at 400 *m/z*, range 300 to 1800 *m/z* was used, and automatic gain control was set to 2 × 10^5^ and maximum inject time to 50 ms. In the linear ion trap, product ion spectra were triggered with a data-dependent acquisition method using “top speed” and “most intense ion” settings. The threshold for collision-induced dissociation (CID) and high energy collisional dissociation (HCD) was set using the Universal Method (above 100 counts, rapid scan rate, and maximum inject time to 10 ms). The selected precursor ions were fragmented sequentially in both the ion trap using CID and in the HCD cell. Dynamic exclusion was set to 30 s. Charge state allowed between +2 and +7 charge states to be selected for MS/MS fragmentation.

Peak lists in format of Mascot generic files (.mgf files) were prepared from raw data using MSConvert package (Matrix Science). Peak lists were searched on Mascot server version 2.4.1 (Matrix Science) against TAIR (version 10) database, a separate in-house constructs database and an in-house contaminants database. Tryptic peptides with up to two possible mis-cleavages and charge states +2, +3, +4 were allowed in the search. The following modifications were included in the search: oxidized Met, phosphorylation on Ser, Thr, Tyr as variable modifications, and carbamidomethylated Cys as a static modification. Data were searched with a monoisotopic precursor and fragment ions mass tolerance 10 ppm and 0.6 Da, respectively. Mascot results were combined in Scaffold version 4 (Proteome Software) and exported to Excel (Microsoft Office).

##### Peptide Binding and Non-denaturing PAGE

Synthetic peptides were obtained from LifeTein (Somerset, NJ) (Table 5). Lyophilized peptides were dissolved in sterile water to a concentration of 1 mg/ml and stored at −20 °C until use. Analysis of peptide binding was carried out following the method described by Arazi *et al.* (46). Briefly, 240 pmol of CaM was incubated with an equal amount of synthetic peptide as indicated in 25 mm Tris-HCl, pH 7.5, containing either 100 μm CaCl_2_ or 2 mm EGTA. Final reaction volumes were 20 μl. Reactions were incubated at room temperature for 60 min after which 12.5 μl of loading buffer was added. Twenty microliters of the sample was then loaded into 12.5% (v/v) acrylamide non-denaturing Tris-glycine gels containing either 100 μm CaCl_2_ or 2 mm EGTA, and samples were separated at 25 mA constant for 90 min. Following electrophoresis, gels were stained with GelCode Blue protein stain.

**TABLE 5:** **Custom peptides used in this study**

Name	Sequence	Molecular mass (Da)	Purpose
W3	LKWKKLLKLLKKLLKLG	2063.8	Binding assays, kinase assays, native PAGE
RG27	RILSKKGNRYSEKDAAVVVRQMLKVAG	3017.62	Native PAGE
FA19	FIKPGKRFHDIVGSAYYVA	2168.55	Native PAGE
RK21	RKPWATISDSAKDFVKKLLVK	2430.94	Native PAGE
FR26	FVKKLLVKDPRARLTAAQALSHAWVR	2975.61	Native PAGE
RR16	RARTAALQALSHAWVR	1807.11	Native PAGE
LL22	LRQFVRYSRLKQFALRALASTL	2638.18	Native PAGE
pLL22	LRQFVRYSRLKQFALRALA(pS)TL*^a^*	2718.18	Native PAGE
ACSM+1	NNLRLSMGKR	1188.41	Kinase substrate

*^a^* This peptide is a modified version of LL22, containing phosphorylation on Ser-360.

##### CPK28 Autophosphorylation and Peptide Kinase Assays

Autophosphorylation assays were carried out using *in situ* dephosphorylated His_6_-CPK28. For time course assays, 20 μg of His_6_-CPK28 was incubated in a 200-μl reaction containing 25 mm Tris-HCl, pH 7.5, 10 mm MgCl_2_, 100 μm CaCl_2_, 1 mm DTT. Reactions were initiated by the addition of 100 μm ATP, and 20 μl of the reaction was withdrawn and stopped by addition to 20 μl of 2× SDS-PAGE loading buffer and heating at 65 °C for 1 min at each time point as indicated. To assess the effect of Ca^2+^ and Ca^2+^/CaM on CPK28 autophosphorylation, reactions were carried out as above but with or without 100 μm CaCl_2_ and with or without 2 μm CaM6 as indicated. Reactions were incubated for 30 min prior to initiation with 100 μm ATP to allow formation of the CPK28-CaM complex. Final reaction volumes were 50 μl and contained 5 μg of His_6_-CPK28. Reactions proceeded for 30 s and were stopped by the addition of 50 μl of 2× SDS-PAGE loading buffer and heating at 65 °C for 1 min. Autophosphorylation reactions were assessed by immunoblotting with anti-Thr(P) and pIMAGO as described above.

Peptide kinase assays were carried out as described previously (40). Briefly, assays contained 40 mm Tris-HCl, pH 7.5, 1 mm DTT, 10 mm MgCl_2_, 100 μm ATP, 0.1 μCi/μl [γ-^32^P]ATP (150 cpm/pmol), 0.5 μg of His_6_-CPK28, and 10 μm ACSM+1 peptide substrate (sequence, NNLRLSMGKR). Reactions were initiated by addition of an ATP/[γ-^32^P]ATP mixture. Additionally, reactions contained combinations of 100 μm CaCl_2_, 0.5 mm EGTA, 1 μm CaM6 or 2 μm W3 peptide as indicated in the appropriate figures. Final reaction volumes were 40 μl. For CaM inhibition experiments, reactions were incubated for 30 min at room temperature prior to addition of ATP. After addition of ATP, reactions were allowed to proceed for 10 min at room temperature and were stopped by spotting 35 μl of each reaction onto P81 phosphocellulose cation exchange paper followed by washing three times for 5 min each in 0.45% (v/v) *o-*phosphoric acid. Incorporation of ^32^P was assessed by liquid scintillation counting.

##### Identification of in Situ CPK28 Autophosphorylation Sites by LC-MS/MS

Purified recombinant P^+^-CPK28 and P^++^-CPK28 were electrophoresed in 10% acrylamide BisTris gels. Gels were fixed in methanol/acetic acid/water (50:7:43), rinsed three times for 10 min each with distilled H_2_O, and stained with Gel Code Blue protein stain (Thermo Fisher Scientific). Protein bands corresponding to CPK28 were excised and minced, and gel pieces were washed with acetonitrile, 100 mm ammonium bicarbonate, pH 8.0 (1:1, v/v), followed by three cycles of shrinking/swelling with acetonitrile and 100 mm ammonium bicarbonate, respectively. Gel pieces were reduced with freshly made 10 mm DTT for 30 min at 37 °C followed by alkylation with 55 mm iodoacetamide for 20 min at room temperature. Gel pieces were subjected to three cycles of shrinking/swelling with acetonitrile and 50 mm ammonium bicarbonate, respectively. The last swelling step included trypsin (Promega Trypsin Gold, Mass Spectrometry grade, catalog no. V5280, lot no. 0000146430) at a concentration of 10 ng/μl. In-gel trypsin digestions were carried out at 37 °C overnight.

CPK28 peptide samples were dried under vacuum and resuspended in 20 μl of 1% acetic acid. Each sample (5 μl each) was subjected to analysis by nano-UPLC using an Easy nanoLC 1000 system (Thermo Fisher Scientific) coupled to an Orbitrap Elite mass spectrometer (Thermo Fisher Scientific). Peptides were separated using a linear gradient from 5% B to 40% B over 60 min (A = 0.1% formic acid, 2% acetonitrile in water; B = 0.1% formic acid in acetonitrile) over a 75-μm inner diameter × 20-cm Picofrit self-pack column (New Objective) packed with 3 μm Magic C18 (Michrom Bioresources) at a flow rate of 300 nl/min. Two sets of data-dependent mass spectrometry data were collected. First, all samples were analyzed using a top five HCD methodology. In a second experiment, samples were subjected to a data-dependent methodology utilizing both HCD and electron transfer dissociation (ETD) in a charge state and mass-to-charge-dependent manner. All +2 charge ions were subjected to HCD, as were +3 ions > *m/z* 650, +4 ions > *m/z* 900, and +5 ions > *m/z* 950. All other ions were subjected to ETD with a 100-ms activation time with supplemental HCD activation enabled. Raw data files were processed using Proteome Discoverer 1.4, and the resulting product ion spectra were searched against the TAIR10 database using Mascot 2.5 with the following parameters: precursor and product ion mass tolerance, 10 ppm; trypsin specificity with up to 1 missed cleavage site; carbamidomethyl-Cys as a fixed modification; phospho-Ser, phospho-Thr, phospho-Tyr, and Met oxidation as variable modifications.

## Author Contributions

K. W. B. wrote the manuscript and designed and carried out all experiments except for *in vivo* analysis of CPK28-interacting proteins by co-immunopurification with CPK28-YFP, which was carried out by J. M., P. D., F. L. H. M., and C. Z. R. K. B. and M. B. G. designed the LC-MS/MS approach for analysis of *in situ* autophosphorylated CPK28. R. E. Z. and S. C. H. conceived the study. Funding was provided by C. Z., M. B. G., R. E. Z., and S. C. H. All authors approved the manuscript prior to submission.

## Supplementary Material

Supplemental Data

## Footnotes

4 The abbreviations used are:

CPK: Ca^2+^-dependent protein kinase
BisTris: 2-[bis(2-hydroxyethyl)amino]-2-(hydroxymethyl)propane-1,3-diol
CaMBD: CaM binding domain
CaM: calmodulin
CaMK: Ca^2+^/CaM-dependent protein kinase
CLD: calmodulin-like domain
CDPK: Ca^2+^-dependent protein kinase
CAD: CDPK activation domain
CLD: CaM-like Ca^2+^-binding domain
IPTG: isopropyl β-d-1-thiogalactopyranoside
HCD: high energy collisional dissociation
ETD: electron transfer dissociation
CID: collision-induced dissociation
CRK: CPK-related kinase
CaMBP: CaM-binding protein
J: junction
CI: confidence interval.
