# Supplementary material for: Autophosphorylation-based Calcium (Ca2+) Sensitivity Priming and Ca2+/Calmodulin Inhibition of Arabidopsis thaliana Ca2+-dependent Protein Kinase 28 (CPK28)
Source: J Biol Chem. 2017 Jan 30;292(10):3988–4002. doi: 10.1074/jbc.M116.763243 (PMC5354511; doi:10.1074/jbc.M116.763243)
Supplement: Supplemental Data [file supp_292_10_3988__index.html]

Autophosphorylation-based calcium (Ca2+) sensitivity priming and Ca2+/Calmodulin inhibition of Arabidopsis thaliana Ca2+-dependent protein kinase 28 (CPK28) — Autophosphorylation-based calcium (Ca2+) sensitivity priming and Ca2+/Calmodulin inhibition of Arabidopsis thaliana Ca2+-dependent protein kinase 28 (CPK28) — Autophosphorylation-based Calcium (Ca2+) Sensitivity Priming and Ca2+/Calmodulin Inhibition of Arabidopsis thaliana Ca2+-dependent Protein Kinase 28 (CPK28) — Regulation of CPK28 by Autophosphorylation and CaM — Supplemental Data 

# Autophosphorylation-based Calcium (Ca2+) Sensitivity Priming and Ca2+/Calmodulin Inhibition of *Arabidopsis thaliana* Ca2+-dependent Protein Kinase 28 (CPK28)

## Supplemental Data

- Supplemental MS data file (.xlsx, 569 KB) - Contains proteins identified by LC/MS/MS in CPK28-YFP immunopurifications.
